# Chemical Profiling of *Re-Du-Ning* Injection by Ultra-Performance Liquid Chromatography Coupled with Electrospray Ionization Tandem Quadrupole Time-of-Flight Mass Spectrometry through the Screening of Diagnostic Ions in MS^E^ Mode

**DOI:** 10.1371/journal.pone.0121031

**Published:** 2015-04-13

**Authors:** Haibo Li, Yang Yu, Zhenzhong Wang, Jianliang Geng, Yi Dai, Wei Xiao, Xinsheng Yao

**Affiliations:** 1 Jiangsu Kanion Pharmaceutical Co. Ltd., Lianyungang, China; 2 Institute of Traditional Chinese Medicine & Natural Products, College of Pharmacy, Jinan University, Guangzhou, China; 3 State Key Lab of New-Tech for Chinese Medicine Pharmaceutical Process, Lianyungang, China; 4 Shenyang Pharmaceutical University, Shenyang, China; University of Glasgow, UNITED KINGDOM

## Abstract

The broad applications and mechanism explorations of traditional Chinese medicine prescriptions (TCMPs) require a clear understanding of TCMP chemical constituents. In the present study, we describe an efficient and universally applicable analytical approach based on ultra-performance liquid chromatography coupled to electrospray ionization tandem quadrupole time-of-flight mass spectrometry (UPLC-ESI-Q/TOF-MS) with the MS^E^ (^E^ denotes collision energy) data acquisition mode, which allowed the rapid separation and reliable determination of TCMP chemical constituents. By monitoring diagnostic ions in the high energy function of MS^E^, target peaks of analogous compounds in TCMPs could be rapidly screened and identified. “Re-Du-Ning” injection (RDN), a eutherapeutic traditional Chinese medicine injection (TCMI) that has been widely used to reduce fever caused by viral infections in clinical practice, was studied as an example. In total, 90 compounds, including five new iridoids and one new sesquiterpene, were identified or tentatively characterized by accurate mass measurements within 5 ppm error. This analysis was accompanied by MS fragmentation and reference standard comparison analyses. Furthermore, the herbal sources of these compounds were unambiguously confirmed by comparing the extracted ion chromatograms (EICs) of RDN and ingredient herbal extracts. Our work provides a certain foundation for further studies of RDN. Moreover, the analytical approach developed herein has proven to be generally applicable for profiling the chemical constituents in TCMPs and other complicated mixtures.

## Introduction

Traditional Chinese medicine prescriptions (TCMPs), which are combinations of several medicinal herbs, have been widely employed for thousands of years in China and other Asian countries. In clinical practice, TCMPs often exhibit significant advantages of low therapeutic risk and remarkable effect for some chronic, multifactorial and systemic diseases [1–4]. However, due to the extreme complexities of multiple TCMP components, revealing their pharmacological material basis and mechanism of action remains challenging. Consequently, an effective and reliable analytical approach for the rapid screening and identification of the multiple components contained in TCMPs is in high demand.

Currently, due to its significant advantages in analytical speed and detection sensitivity, ultra-performance liquid chromatography coupled with electrospray ionization tandem quadrupole time-of-flight mass spectrometry (UPLC-ESI-Q/TOF-MS) has become an irreplaceable technique for the on-line structural elucidation of multiple components in mixtures, especially for complex TCMs/TCMPs, biological samples and pesticide residues [5–9]. UPLC coupled with MS^E^ (^E^ represents collision energy) technology provides an automated strategy to decrease analysis time and maximize duty cycles by using parallel alternating scans at low collision energy in the collision cell to obtain precursor ion information or at high collision energy to obtain accurate full-scan mass fragment, precursor ion and neutral loss information. Therefore, both precursor and fragmentation data in exact mass mode were collected in a single run; this method has provided excellent chromatographic and MS efficiencies for the rapid structural elucidation of multiple constituents in complex mixtures [10–12]. In the present study, based on the point of view that a certain type of chemical compounds could produce identical or similar characteristic fragment ions under a suitable collision energy in their tandem mass spectra, a well-designed analytical approach that enabled rapid screening and characterization of multiple TCMP constituents was developed. By virtue of UPLC-ESI-Q/TOF-MS and optimized MS^E^ method, diagnostic fragment ions can be used as invaluable evidence for the detection of both expected and unexpected chemical constituents within TCMPs.

Re-Du-Ning injection (RDN), a traditional Chinese medicine injection (TCMI), was manufactured by Jiangsu Kanion Pharmaceutical Co. Ltd. (Lianyungang, China) and consists of three common herbs: *Lonicera japonica* Thunb. (*L*. *japonica* Thunb.; Jin-yin-hua), *Gardenia jasminoides* Ellis (*G*. *jasminoides* Ellis; Zhi-zi) and *Artemisia annua* L. (*A*. *annua* L.; Qing-hao). In China, RDN is widely used for the treatment of viral infection, such as hand-foot-mouth disease [13–14], influenza [15] and herpes angina efficacy [16]. Although RDN has proven to be clinically effective, the knowledge of its chemical constituents is still limited. The elucidation of the various components contained in RDN is urgently necessary and of great importance to RDN quality control and to understanding its mechanism of action.

In this paper, a robust Waters UPLC-ESI-Q/TOF-MS system and optimized MS^E^ method was utilized, employing RDN as an example for illustration. To our knowledge, this work is the first study on the chemical components contained in RDN using the methodology developed herein. As a result, a total of 90 compounds, including 45 iridoids, 21 organic acids, nine flavonoids, seven lignans, four sesquiterpenes, three coumarins and one monoterpene were identified or tentatively characterized in RDN. In addition, the source plants of these compounds were confirmed by comparing the extracted ion chromatograms (EICs) of RDN to the corresponding ingredient herbs. This work provides a certain foundation for further studies of RDN. More importantly, this novel approach is expected to be widely applied for analyzing other TCMPs and complex mixtures.

## Materials and Methods

### 2.1. Chemicals and materials

*G*. *jasminoides* Ellis, *L*. *japonica* Thunb. and *A*. *annua* L. were purchased from the Ji'an Medical Material Market (Jiangxi, China). All herbal medicines were identified by Professor Zhou Wu (Jiangsu Kanion Pharmaceutical Co. Ltd.). A voucher specimen was deposited in Jiangsu Kanion Pharmaceuticals (Lianyungang, China). The Re-Du-Ning injection (Batch number: 100906) was manufactured and supplied by Jiangsu Kanion Pharmaceutical Co. Ltd. (Lianyungang, China).

All reference standards were isolated from the RDN injection by various column chromatography techniques and were unambiguously identified by nuclear magnetic resonance (NMR) and MS methods in our laboratory.

Liquid chromatography (LC)-MS-grade acetonitrile and water were purchased from Fisher Scientific (Fair Lawn, New Jersey, USA). LC-MS-grade formic acid was obtained from Sigma-Aldrich (St. Louis, USA). The water, methanol and ethanol used for sample extraction were all of analytical grade.

### 2.2. Sample preparation

The RDN samples were directly evaporated with a rotary evaporator and then diluted to 10 mg/mL. Next, 2 mL of these solutions were transferred into separate clean tubes and dried under nitrogen gas at room temperature. The residues were reconstituted in 2 mL of water and then centrifuged at 10000 rpm for 10 min. Solid-phase extraction (SPE) cartridges, (Vac 3cc, 200 mg, Phenomenex strata C18-E, Torrance, CA) were preconditioned with 3 mL of methanol, followed by 3 mL of water before use. The supernatants were loaded onto the SPE cartridges and washed with 2 mL of water. The SPE cartridges were then eluted with 4 mL of methanol, and the eluents were centrifuged at 10000 rpm for 10 min. Supernatant aliquots of 2 μL were injected into the UPLC/Q-TOF-MS system for analyses.

Ingredient herbal medicine samples (*G*. *jasminoides*, 2 g; *L*. *japonica*, 2 g; *A*. *annua*, 2 g) were immersed in 20 mL of deionized water for 1 h. The solutions were then decocted by boiling 3 times (1 h each time). The extracts were diluted to generate 20 mg/mL solutions. All samples were filtered through a 0.22 μm filter membrane before UPLC-MS analyses.

### 2.3. UPLC-Q/TOF-MS analyses

UPLC analyses were performed using an ACQUITY UPLC system equipped with a binary solvent system, an automatic sample manger and photodiode array (PDA) detector. The chromatographic separation was performed on an Acquity UPLC BEH C18 Column (3.0 mm × 150 mm, 1.7 μm, waters, Ireland) at a temperature of 40°C. The mobile phases consisted of eluent A (0.1% formic acid in water, v/v) and eluent B (0.1% formic acid in acetonitrile, v/v). These eluents were delivered at a flow rate of 0.4 mL/min with a linear gradient program as follows: 2–5% B from 0 to 5.0 min, 5–12% B from 5.0 to 10.0 min, 12–30% B from 10.0 to 15.0 min, 30–55% B from 15.0 to 19.0 min and 55–100% B from 19.0 to 20.0 min. After maintaining 100% B for 3 min, the column was returned to its initial condition.

The UPLC system was coupled to a hybrid quadrupole, orthogonal time-of-flight (Q-TOF) tandem mass spectrometer (SYNAPT G2 HDMS, Waters, Manchester, U.K.) equipped with ESI. The operating parameters were as follows: capillary voltage of 3 kV (ESI+) or -2.5 kV (ESI-), sample cone voltage of 35 V, extraction cone voltage of 4 V, source temperature of 100°C, desolvation temperature of 300°C, cone gas flow of 50 L/h and desolvation gas flow of 800 L/h. In MS^E^ mode, the trap collision energy for the low-energy function was set at 5 eV, while the ramp trap collision energy for the high-energy function was set at 20–50 eV. Argon was used as the collision gas for collision-induced dissociation (CID) in MS^E^ and MS^2^ modes. To ensure mass accuracy and reproducibility, the mass spectrometer was calibrated over a range of 50–1500 Da using a solution of sodium formate. Leucine-enkephalin (*m/z* 556.2771 in positive ion mode; *m/z* 554.2615 in negative ion mode) was used as an external reference for the LockSpray and was infused at a constant flow of 5 μL/min. The data were centroided during acquisition.

## Results and Discussion

### 3.1. Optimization of UPLC and mass spectrometry conditions

The MS^E^ acquisition mode required well-resolved peaks to ensure that the predominant fragments were collected from a single precursor ion. Obtaining a desirable chromatographic profile with satisfactory separation and peak shapes without excessive peak tailing was necessary. Different UPLC conditions that included both mobile phase systems (methanol-aqueous and acetonitrile-aqueous) were tested. When the mobile phase was acetonitrile-aqueous, the separation resolution was greatly improved compared to methanol-aqueous. Addition of 0.1% formic acid to the mobile phase reduced peak tailing and enhanced the resolution. Thus, an acetonitrile-aqueous solution with 0.1% formic acid was selected as the mobile phase. In addition, four analytical columns, including the Acquity BEH C18 column (3.0 mm × 150 mm, 1.7 μm), Acquity BEH C18 column (2.1 mm × 50 mm, 1.7 μm), Acquity HSS T3 column (2.1 mm × 50 mm, 1.8 μm) and Acquity Shield PR18 column (2.1 mm × 50 mm, 1.7 μm) were compared to achieve better separation performance. Unfortunately, the results of three different 50 mm columns were not satisfactory as shown in (S1 Fig). Then, we found that the column length could influence the separation efficiency significantly. Thus, the Acquity BEH C18 column (3.0 mm × 150 mm, 1.7 μm) was chosen for analysis in current condition. For mass spectrometry, both positive and negative ion modes were tested, and each target compound type was analyzed in a suitable ESI mode.

### 3.2. Establishment of the supporting database

A systematic investigation of the chemical constituents in RDN was conducted. A self-built database of compounds that were isolated from three medicinal herb ingredients of RDN was established by retrieving on-line databases or Internet search engines, such as Chemical Abstracts Service (CAS) database, Massbank, Web of Science and ChemSpider. The emphasis was placed on analyzing structural characteristics and MS fragmentation behaviors, especially for diagnostic ions (characteristic fragments). As a result, 259 constituents, including iridoids, organic acids, flavonoids, sesquiterpenes, lignans and coumarins were collected. Five items, compound name, molecular formula, accurate mass, diagnostic fragment ions or neutral losses and UV absorption, were recorded.

### 3.3. Diagnostic ion screening using the optimized MS^E^ method

All chemical constituents in herbal medicine can be categorized into different families based on structural types. Thus, a certain family of compounds with identical carbon skeletons could produce similar characteristic fragment ions under CID conditions in mass spectrometry.

Accordingly, the core idea of our approach is to use the diagnostic ions as markers for target compound detection. To simultaneously generate both precursor and fragment ions using the MS^E^ method, low- and high-energy scan functions were switched rapidly and continuously for data acquisition. The high-energy scan function that is used to collect information on fragment ions is generally equivalent to a non-selective MS/MS scan. With such a function, specific diagnostic ions of diverse compounds contained in TCMPs and their precursor ions and neutral losses were simultaneously collected, providing large quantities of valuable information regarding the structural identification of chemical constituents.

**Fig 1:**
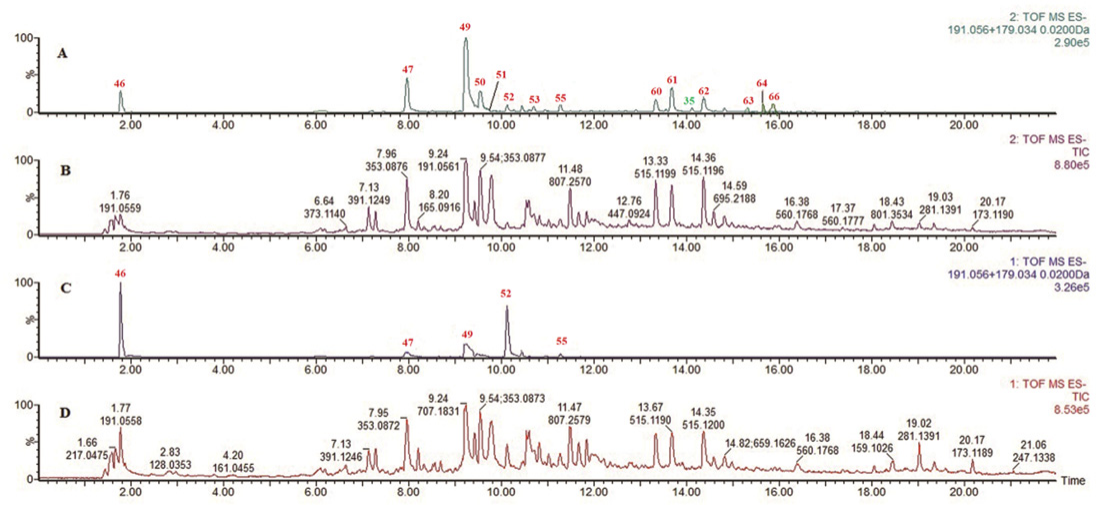
MS chromatograms of diagnostic ions: (A) EICs of diagnostic ions 179.0340 and 191.0556 in the high-energy function of MS^E^; (B) TIC of RDN in the high-energy function of MS^E^; (C) EICs of diagnostic ions 179.0340 and 191.0556 in the low-energy function of MS^E^; (D) TIC of RDN in the MS^E^ low-energy function.

In this study, the screening process of caffeoylquinic acids was considered to describe the approach in detail. Based on the aforementioned self-built database, fragment ions at *m/z* 191.0556 and *m/z* 179.0340, which can be produced from caffeoylquinic acids as common sub-structures, were selected as diagnostic ions for detecting other analogues. As shown in (Fig 1), the peaks that appeared in the EIC of the high-energy function of the MS^E^ mode were considered as target compounds and further characterized by accurate mass measurements, MS fragmentation analyses and reference standards. Interestingly, an unexpected compound (labeled as **35**) that possessed a novel structure with a rare caffeoylquinic ester acylated at the C-10 position of geniposide was similarly screened out. By comparison, only a few peaks could be detected in the EIC mode of the low-energy MS^E^ function. A wide range of ramped CE (20–50 eV in the present study) in the high-energy MS^E^ function will help reveal the MS fragmentation behaviors of different compounds simultaneously. Similarly, other types of analogues were rapidly screened out by our proposed approach, such as iridoids (S2 Fig), flavonoids (S3 Fig) and others.

### 3.4. Identification of chemical constituents in RDN

**Fig 2:**
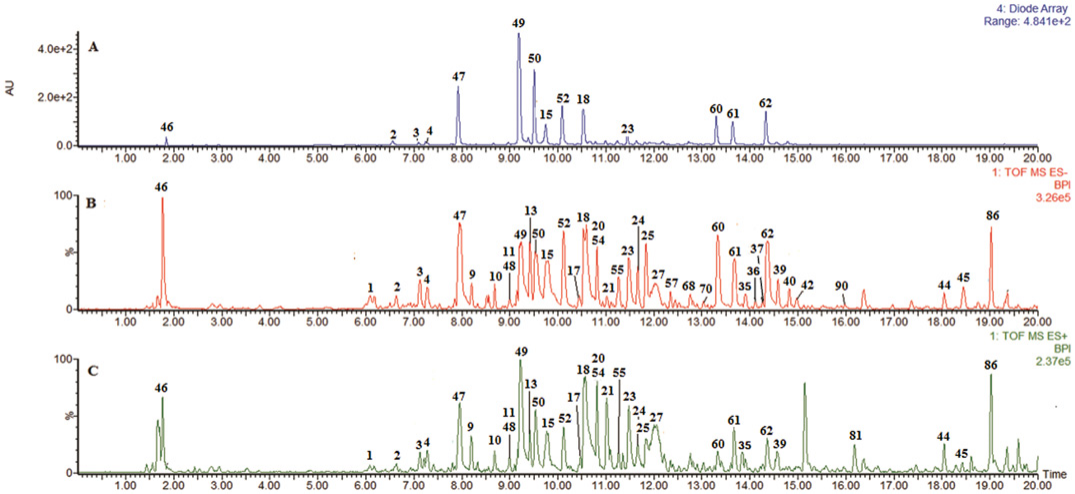
UPLC-ESI-Q-TOF-MS analysis of RDN: (A) UV (225 nm) chromatograph; (B) (-) ESI-MS BPI profile; (C) (+) ESI-MS BPI profile.

A total of 90 compounds, including 45 iridoids, 21 organic acids, nine flavonoids, seven lignans, four sesquiterpenes, three coumarins and one monoterpene were identified or tentatively characterized in RDN (Table 1; S4 Fig). The herb sources of these compounds were confirmed by comparing the base peak chromatograms of RDN to a single herbal extract. The main active constituents of RDN (i.e., caffeoylquinic acids and iridoids) were rapidly screened out by UPLC-ESI/Q-TOF mass spectrometry through diagnostic ion screening with MS^E^. The remaining compounds were identified according to their accurate mass measurements within 5 ppm error, tandem MS behaviors, database-matching and reference standards. Both negative and positive ion modes were examined, and the base peak intensity (BPI) profiles of RDN and three ingredient herbs are shown in (Fig 2, S5 and S6 Figs). Recently, ultra performance liquid chromatography coupled with quadrupole time-of-flight mass spectrometry (UPLC-Q-TOF-MS) has been widely used to characterize chemical profiling of herbal medicines and TCMPs. This method has become one of the most frequently applied approaches in the area of fast chromatographic separations [17–19]. High-resolution tandem mass spectrometry can provide a more specific and accurate mass as long as the co-eluting compounds possess different *m/z* values [20]. And the isotopic abundances and the elemental composition of fragment ions are greatly conducive to the structural elucidation of unknown compounds. However, it should be pointed out that identification of chemical components from complex TCMPs relying solely on mass spectrometry-based approaches was insufficient. As the spectral differences for some isomers are very small and they cannot be differentiated and unambiguously identified. Therefore, in present study, some reference standards isolated from the entitled injection were used to validate the elucidation of those isomers. Thus, it provides the enhanced accuracy and reliability of MS quantitative results.

**Table 1:** Compounds identified in RDN by UPLC-ESI-Q-TOF-MS.

No	t_*R*_	Selected ion	Elemental composition	Measured mass	Calculated mass	Mass error	MS^E^ or MS^2^ fragmentation	Identification	Source^a^
1	6.30	[M+Na]^+^	C_16_H_22_O_11_	413.1056	413.1060	-1.0	251.0533, 233.0425;	deacetylasperulosidic acid	Gj
2	6.64	[M+Na]^+^	C_16_H_22_O_10_	397.1111	397.1111	0.0	235.0584, 217.0475;	gardoside	Gj
3	7.12	[M+Na]^+^	C_16_H_24_O_11_	415.1216	415.1216	0.0	253.0685, 235.0581, 217.0478, 173.0579;	shanzhiside	Gj
4	7.28	[M+Na]^+^	C_16_H_22_O_10_	397.1111	397.1111	0.0	235.0582, 217.0477, 173.0578;	geniposidic acid	Gj
5	7.53	[M+Na]^+^	C_16_H_22_O_11_	413.1064	413.1060	1.0	251.0537, 233.0423, 215.0322;	monotropein	GJ
6	7.73	[M+Na]^+^	C_17_H_24_O_11_	427.1200	427.1216	-3.7	265.0687, 247.0583, 215.0324;	deacetylasperulosidic acid methyl ester	Gj
7	7.80	[M+Na]^+^	C_17_H_26_O_11_	429.1363	429.1373	-2.3	267.0844, 249.0738, 217.0476;	shanzhiside methyl ester	Gj
8	7.86	[M+Na]^+^	C_16_H_22_O_11_	413.1073	413.1060	3.1	251.0536, 233.0422;	scandoside	Gj
9	8.30	[M+Na]^+^	C_17_H_24_O_11_	427.1218	427.1216	0.5	265.0687, 247.0583, 215.0324;	gardenoside	Gj
10	8.68	[M+Na]^+^	C_17_H_24_O_10_	411.1263	411.1261	1.0	249.0738, 231.0634, 199.0370;	8-epi-apodantheroside	Gj
11	9.01	[M+Na]^+^	C_16_H_22_O_12_	429.1025	429.1009	2.1	267.0485, 249.0378, 217.0115;	8-epi-kingiside	Lj
12	9.18	[M+Na]^+^	C_17_H_26_O_11_	429.1369	429.1373	-0.9	267.0846, 249.0741, 217.0479;	morroniside	Lj
13	9.42	[M+Na]^+^	C_16_H_22_O_11_	413.1058	413.1060	-0.5	251.0541, 233.0427;	secologanoside	Lj
14*	9.56	[M+Na]^+^	C_23_H_34_O_15_	573.1782	573.1795	-2.3	411.1268, 249.0740, 231.0633, 199.0372;	genipin-1-β-d-gentiobioside	Gj
15	9.78	[M+Na]^+^	C_16_H_22_O_10_	397.1115	397.1111	1.0	235.0584, 217.0475, 199.0371, 173.0214,147.0056;	secologanic acid	Lj
16	9.90	[M+Na]^+^	C_16_H_22_O_12_	429.1010	429.1009	0.1	267.0487, 249.0379, 217.0111;	kingiside	Lj
17	10.48	[M+Na]^+^	C_17_H_26_O_10_	413.1429	413.1424	1.2	251.0894, 233.0788;	loganin	Lj
18*	10.56	[M+Na]^+^	C_17_H_24_O_10_	411.1279	411.1267	2.9	249.0741, 209.0829, 199.0372;	geniposide	Gj
19*	10.57	[M+H]^+^	C_28_H_36_O_14_	597.2195	597.2183	2.7	435.1654, 417.1548, 207.0654, 175.0397;	jasmigeniposide B	Gj
20	10.81	[M+Na]^+^	C_16_H_22_O_9_	381.1156	381.1162	-1.6	219.0632, 201.0525, 173.0577;	sweroside	Lj
21	11.02	[M+Na]^+^	C_17_H_22_O_10_	409.1100	409.1111	-2.7	247.0583, 229.0478;	methyl1-(β-d-glucopyranosyloxy)-7-(hydroxymethyl)-1,7a-dihydrocyclopenta[c]pyran-4-carboxylate	Gj
22*	11.10	[M+Na]^+^	C_25_H_28_O_12_	543.1472	543.1478	-1.1	397.1114, 235.0585, 217.0482;	6′-*O*-*trans*-p-coumaroylgeniposidic acid	Gj
23*	11.51	[M+Na]^+^	C_17_H_24_O_11_	427.1207	427.1216	-2.1	265.0674, 247.0590, 215.0326;	secoxyloganin	Lj
24*	11.66	[M+Na]^+^	C_17_H_24_O_10_	411.1266	411.1267	-0.2	249.0741, 231.0642, 199.0374;	7-epi-vogeloside	Lj
25*	11.84	[M+Na]^+^	C_17_H_24_O_10_	411.1265	411.1267	-0.5	249.0739, 231.0644, 199.0378;	vogeloside	Lj
26*	11.93	[M+H]^+^	C_25_H_33_NO_11_	524.2131	524.2132	-0.2	362.1603;	L-phenylalanino secologanin	Lj
27*	12.04	[M+Na]^+^	C_17_H_24_O_10_	411.1269	411.1267	0.5	249.0745, 231.0642, 199.0377;	secologanin	Lj
28	12.35	[M+Na]^+^	C_19_H_26_O_11_	453.1388	453.1373	3.3	411.1266, 249.0741, 217.0478;	6′-*O*-acetylgeniposide	Gj
29	13.03	[M+H]^+^	C_20_H_27_NO_11_	458.1682	458.1662	4.4	296.1133, 278.1029;	lonijaposide J	Lj
30	13.11	[M+Na]^+^	C_18_H_26_O_11_	441.1375	441.1373	0.5	279.0844, 261.0744, 229.0472;	dimethyl secologanoside	Lj
31	13.21	[M+Na]^+^	C_27_H_32_O_14_	603.1688	603.1690	-0.3	422.1191, 260.0663, 242.0554;	6′-*O*-*trans*-sinapoyl gardoside	Gj
32	13.72	[M+H]^+^	C_18_H_25_NSO_8_	416.1372	416.1379	-1.7	254.0852, 236.0746;	xylostosidine	Lj
33	13.80	[M+Na]^+^	C_21_H_28_O_13_	511.1425	511.1428	-0.4	411.1254, 249.0742, 231.0712;	10-*O*-succinoylgeniposide	Gj
34	13.83	[M+H]^+^	C_19_H_25_NSO_10_	460.1281	460.1277	0.9	298.0751, 280.0646;	xylostosidine	Lj
35*	14.11	[M+Na]^+^	C_33_H_40_O_18_	747.2125	747.2112	1.7	377.0778, 585.1559, 553.1147, 535.1148, 411.1322, 393.070, 231.1102, 199.0407, 215.0157;	jasmigeniposide A	Gj
36	14.22	[M+Na]^+^	C_19_H_30_O_11_	457.1685	457.1686	-0.1	295.1157, 277.1054, 263.0895;	secologanin dimethyl acetal	Lj
37*	14.26	[M+Na]^+^	C_34_H_46_O_19_	781.2532	781.2531	0.1	619.2036, 549.1576, 517.1330, 387.1042, 355.0804;	*(Z)*-aldosecologanin	Lj
38	14.35	[M+H]^+^	C_25_H_28_O_12_	521.1658	521.1659	-0.1	375.1294, 213.0764, 195.0659;	2′-*O*-p-hydroxybenzoyl gardoside	Gj
39	14.58	[M+Na]^+^	C_32_H_40_O_17_	719.2181	719.2163	2.5	511.1414, 493.1324, 209.0816;	6″-*O*-*trans*-*p*-coumaroylgenipin gentiobioside	Gj
40*	14.75	[M+Na]^+^	C_34_H_44_O_19_	779.2378	779.2374	0.5	571.1626, 553.1534, 209.0830;	6″-*O*-*trans*-sinapoylgenipin gentiobioside	Gj
41*	14.84	[M+Na]^+^	C_34_H_46_O_19_	781.2533	781.2531	0.3	619.2133, 549.1332, 517.1320, 387.1041, 355.0841;	*(E)*-aldosecologanin	Lj
42*	14.97	[M+Na]^+^	C_33_H_42_O_18_	749.2266	749.2269	-0.4	541.1472, 523.1427, 209.0824;	6″-*O-trans-p*-feruloylgenipin gentiobioside	Gj
43	16.33	[M+Na]^+^	C_28_H_34_O_14_	617.1845	617.1846	-0.2	411.1267, 249.0801, 231.0639, 199.0382;	6′-*O*-*trans*-sinapoylgeniposide	Gj
44*	18.04	[M+H]^+^	C_25_H_31_NO_10_	506.2032	506.2026	1.2	344.1493, 326.1419, 298.1437, 274.1083, 256.1046, 228.6037;	L-phenylalanino secologanin B	Lj
45*	18.45	[M+Na]^+^	C_32_H_40_O_16_	703.2203	703.2214	-1.6	495.1643, 477.1374, 209.0827;	6″-*O*-*trans*-cinnamoylgenipin gentiobioside	Gj
46	1.77	[M-H]^-^	C_7_H_12_O_6_	191.0561	191.0556	2.6	173.0466, 137.0236, 129.0551;	quinic acid	Lj/Gj
47*	7.95	[M-H]^-^	C_16_H_18_O_9_	353.0876	353.0873	0.8	191.0559; 179.0439; 129.0553, 161.0235, 135.0452;	5-*O*-caffeoylquinic acid	Lj/Gj/Aa
48	9.00	[M-H]^-^	C_7_H_6_O_3_	137.0238	137.0239	-0.7	Overlapped in MS^E^ chromatogram	salicylic acid	Lj/Gj/Aa
49*	9.23	[M-H]^-^	C_16_H_18_O_9_	353.0879	353.0873	1.7	191.0560, 179.0357, 173.0469,161.0265, 135.0458;	3-*O*-caffeoylquinic acid	Lj/Gj/Aa
50*	9.54	[M-H]^-^	C_16_H_18_O_9_	353.0875	353.0873	0.6	191.0561, 179.0352, 173.0454, 161.0217, 135.0450;	4-*O*-caffeoylquinic acid	Lj/Gj/Aa
51*	9.72	[M-H]^-^	C_17_H_20_O_9_	367.1036	367.1029	1.9	353.0876, 191.0549, 179.0380, 173.0411, 161.0265, 135.0424;	5-*O*-caffeoylquinic methyl ester	Lj/Gj/Aa
52*	10.12	[M-H]^-^	C_9_H_8_O_4_	179.0345	179.0344	0.3	135.0445;	*trans*-caffeic acid	Lj/Gj
53*	10.33	[M-H]^-^	C_17_H_20_O_9_	367.1046	367.1029	4.6	353.0786, 191.0527, 179.0388, 173.0450, 161.0259, 135.0468;	3-*O*-caffeoylquinic methyl ester	Lj/Gj/Aa
54	10.81	[M+H]^+^	C_10_H_12_O_4_	197.0813	197.0814	-0.5	Overlapped in MS^E^ chromatogram	3-hydroxy-4-methoxy styrene acrylic acid	Lj/Gj
55*	11.26	[M-H]^-^	C_17_H_20_O_9_	367.1042	367.1029	3.5	353.0871, 191.0565, 179.0366, 173.0456, 161.0313; 135.0445;	4-*O*-caffeoylquinic methyl ester	Lj/Gj
56	11.47	[M+H]^+^	C_9_H_8_O_3_	165.0547	165.0552	-3.0	Overlapped in MS^E^ chromatogram	*trans*-p-hydroxycinnamic acid	Lj/Gj
57	11.94	[M-H]^-^	C_16_H_16_O_8_	335.0768	335.0767	1.0	179.0353;	3-*O*-caffeoylshikimic acid	Gj
58*	12.21	[M+H]^+^	C_9_H_8_O_2_	149.0604	149.0603	0.7	Overlapped in MS^E^ chromatogram	*trans*-cinnamic acid	Lj
59	13.25	[M-H]^-^	C_9_H_10_O_4_	181.0500	181.0501	-0.3	Overlapped in MS^E^ chromatogram	syringaldehyde	Gj/Aa
60*	13.33	[M-H]^-^	C_25_H_24_O_12_	515.1201	515.1190	2.1	353.0887, 191.0567, 179.0357, 173.0456, 161.0255, 135.0455;	3,4-di-*O*-caffeoylquinic acid	Lj/Gj
61*	13.68	[M-H]^-^	C_25_H_24_O_12_	515.1190	515.1190	0	353.0875, 191.0561, 179.0349, 173.0446, 161.0266, 135.0454;	3,5-di-*O*-caffeoylquinic acid	Lj/Gj
62*	14.35	[M-H]^-^	C_25_H_24_O_12_	515,1197	515.1190	1.4	353.0881, 191.0562, 179.0356, 173.0455, 161.0251, 135.0455;	4,5-di-*O*-caffeoylquinic acid	Lj/Gj
63*	15.29	[M-H]^-^	C_26_H_26_O_12_	529.1360	529.1346	2.6	367.1013, 349.0910, 179.0375, 161.0289, 135.0448;	3,4-di-O-caffeoylquinic methyl ester	Lj/Gj
64*	15.62	[M-H]^-^	C_26_H_26_O_12_	529.1361	529.1346	2.8	367.1048, 349.0920, 179.0344, 161.0319, 135.0466;	3,5-di-*O*-caffeoylquinic methyl ester	Lj/Gj
65	15.67	[M-H]^-^	C_10_H_10_O_4_	193.0500	193.0501	-0.7	Overlapped in MS^E^ chromatogram	*trans*-ferulic acid	Lj/Gj/Aa
66*	15.80	[M-H]^-^	C_26_H_26_O_12_	529.1356	529.1346	1.9	367.1040, 349.0918, 179.0352, 161.0326, 135.0434;	4,5-di-*O*-caffeoylquinic methyl ester	Lj/Gj
67	12.10	[M-H]^-^	C_27_H_30_O_16_	609.1461	609.1456	0.8	301.0346, 283.0244, 181.0138;	rutin	Lj/Gj
68	12.53	[M-H]^-^	C_21_H_20_O_12_	463.0880	463.0877	0.6	301.0347, 181.0140;	hyperoside	Lj/Gj
69	12.67	[M-H]^-^	C_27_H_30_O_15_	593.1501	593.1506	-0.8	447.0928, 285.0398;	lonicerin	Lj/Gj
70*	12.76	[M-H]^-^	C_21_H_20_O_11_	447.0933	447.0927	1.3	285.0399, 165.0197;	luteolin-7-*O*-β-d-glucoside	Lj/Gj
71	12.77	[M-H]^-^	C_15_H_12_O_6_	287.0556	287.0556	0	125.0238;	eriodictyol	Lj
72	13.59	[M-H]^-^	C_20_H_20_O_8_	387.1086	387.1080	1.5	236.0686;	artemetin	Aa
73*	17.53	[M-H]^-^	C_15_H_10_O_6_	285.0402	285.0399	1.0	267.0295, 239.0344, 165.0195;	luteolin	Gj
74*	17.63	[M-H]^-^	C_15_H_10_O_7_	301.0350	301.0348	0.2	283.0245, 255.0295, 181.0140, 155.0346;	quercetin	Lj/Gj/Aa
75	20.00	[M-H]^-^	C_18_H_16_O_8_	359.0765	359.0767	-0.5	208.0371;	eupatin	Aa/Gj
76	11.88	[M-H]^-^	C_28_H_38_O_13_	581.2239	581.2234	0.9	419.1705, 387.1443, 355.1183;	lyoniresinol-9-*O*-β-d-glucopyranoside	Gj
77	13.01	[M-H]^-^	C_22_H_28_O_8_	419.1700	419.1706	-1.4	387.1448, 355.1184;	lyoniresinol	GJ/Lj
78*	13.11	[M+Na]^+^	C_20_H_26_O_7_	401.1581	401.1576	1.2	Identified by standard compound	threo-1-(4-hydroxy-3-methoxyphenyl)-2-[2-hydroxy-4-(3-hydroxypropyl) phenoxy]-1,3-propanediol	Aa
79*	13.40	[M+Na]^+^	C_20_H_26_O_7_	401.1590	401.1576	3.5	Identified by standard compound	erythro-1-(4-hydroxy-3-methoxyphenyl)-2-[2-hydroxy-4-(3-hydroxypropyl) phenoxy]-1,3-propanediol	Aa
80	15.51	[M+H]^+^	C_21_H_26_O_7_	391.1700	391.1757	3.3	359.1496;	3,3′,5-trimethoxy-4′,7-epoxy-8,5′-neolignan-4,9,9′-triol	Lj
81	16.18	[M-H]^-^	C_20_H_24_O_6_	359.1512	359.1495	4.8	327.1233;	dihydrodehydrodiconiferyl alcohol	Lj
82	16.64	[M-H]^-^	C_20_H_20_O_6_	355.1183	355.1182	0.3	Identified by standard compound	balanophonin	Gj
83	12.81	[M-H]^-^	C_21_H_34_O_9_	429.2121	429.2125	-0.9	267.1596, 249.1490, 231.1383;	(1*R*,7*R*,8*S*,10*R*)-7,8,11-trihydroxyguai-4-en-3-one 8-*O*-β*-* d-glucopyranoside	Gj
84*	17.46	[M+Na]^+^	C_15_H_20_O_4_	287.1247	287.1259	-4.2	243.1362;	*Z-*abscisic acid	Lj
85*	17.66	[M+Na]^+^	C_15_H_20_O_4_	287.1250	287.1259	-3.1	243.1363;	*E-*abscisic acid	Lj
86*	19.02	[M-H]^-^	C_15_H_22_O_5_	281.1394	281.1389	1.8	237.1490, 193.1590;	(1*S*,6*R*,7*R*,10*R*)-6-carboxy-10-methyl-α-methylene-1-(1-oxobutyl)- cyclohexaneacetic acid	Aa
87	12.74	[M+H]^+^	C_10_H_8_O_4_	193.0509	193.0501	4.1	165.0554, 139.0394;	scopoletin	Gj/Aa
88*	13.15	[M+H]^+^	C_11_H_10_O_5_	223.0609	223.0606	1.3	195.0656, 169.0500;	7-hydroxy-6,8-dimethoxyphenyl coumarin	Aa
89*	14.58	[M+H]^+^	C_9_H_6_O_2_	147.0447	147.0446	0.7	119.0498, 96.0342;	coumarin	Gj
90*	15.82	[M-H]^-^	C_21_H_34_O_11_	461.2019	461.2023	-0.9	329.1602, 167.1070, 123.1177;	(2*E*,6*S*)-8-[α-L-arabinopyranosyl-(1"-6')-β-d-glucopyranosyloxy]-2, 6-dimethylct-2-eno-1,2"-lactone	Lj

^a^ *Gardenia jasminoides* Ellis, *Lonicera japonica* Thunb. and *Artemisia annua* are abbreviated as Gj, Lj and Aa, respectively;

* This compounds were identified by standard compounds;

### 3.4.1. Iridoids

Iridoids are the main constituents of RDN. This category of compounds was primarily derived from *L*. *japonica* and *G*. *jasminoides*. In this study, 45 iridoids were identified in positive ion mode. Four of the iridoids were new compounds, which were previously isolated and identified using NMR [21]. The diagnostic fragment ions of these compounds were previously reported [22–24]. Such fragments include the neutral cleavage of the glycosidic bond with the neutral loss of a glucose unit (162 Da) and subsequent losses of H_2_O, CO and CH_3_OH. As shown in (S2 Fig), 32 peaks appearing in EIC mode were considered as target compounds by extracting the diagnostic ions 209.0814, 251.0532, 235.0582 and 215.0320 with the high-energy MS^E^ function. The proposed fragmentation pathways of typical compounds are discussed in detail below.

**Fig 3:**
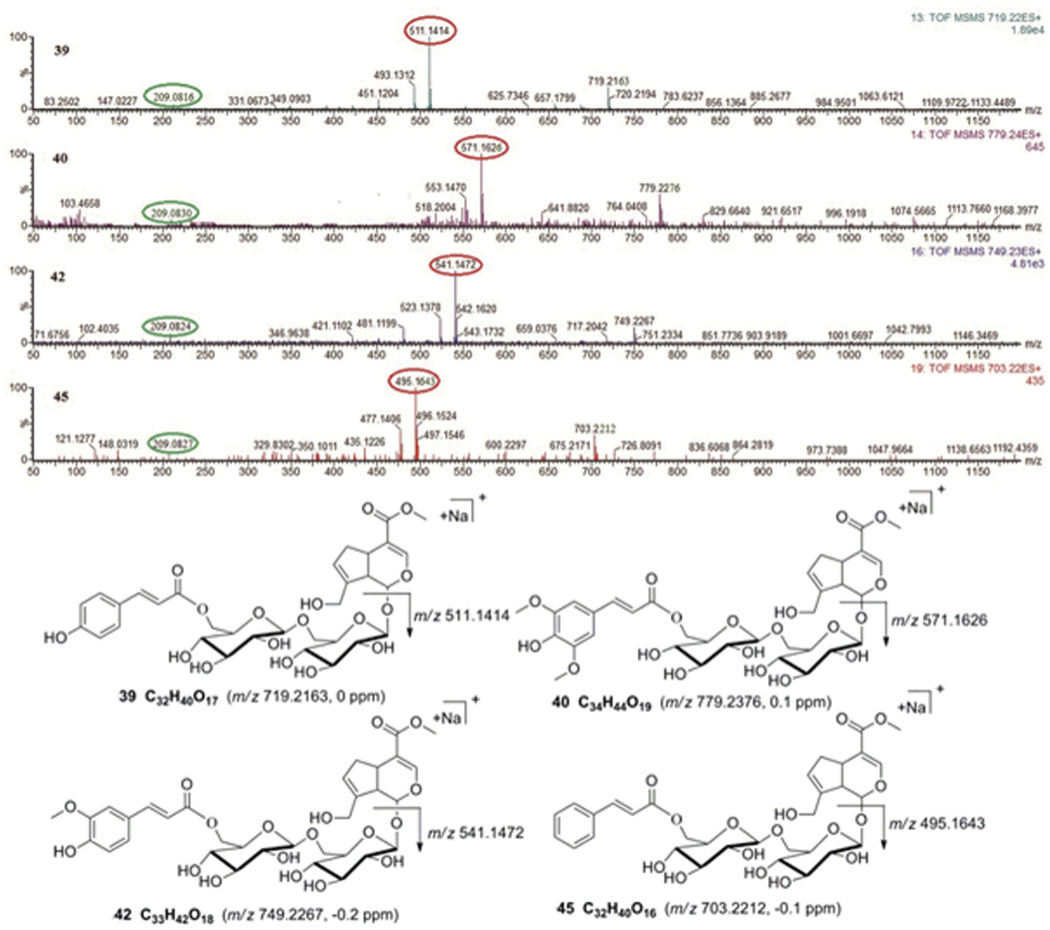
MS/MS spectra and proposed fragmentation pathways of compounds 39, 40, 42 and 45.

Compounds **39**, **40**, **42** and **45** were unambiguously identified as 6″-*O*-*trans*-*p*-coumaroylgenipin gentiobioside, 6″-*O*-*trans*-sinapoylgenipin gentiobioside, 6″-*O-trans-*feruloylgenipin gentiobioside and 6″-*O*-*trans*-cinnamoylgenipin gentiobioside, respectively, by comparing their retention times with authentic reference substances isolated from RDN and fragmentation pathways observed in the MS/MS experiments. Of these, compound **42** was new. Interestingly, we discovered that the MS/MS spectra of their [M+Na]^+^ adducts showed base peaks at *m/z* 511.1414 (C_21_H_28_O_13_Na), 571.1626 (C_23_H_32_O_15_Na), 541.1472 (C_22_H_30_O_14_Na) and 495.1643 (C_21_H_28_O_12_Na), respectively. All of these peaks were produced by the loss of a C_11_H_12_O_4_ fragment (Fig 3). This fragmentation pathway is different from that of iridoid glycosides in which the C6-C3 unit is not substituted onto the C-6 position of the glucose unit. We presumed that the C6-C3 unit, an electron-donating group, might have led to this phenomenon. This mechanism should be further investigated.

**Fig 4:**
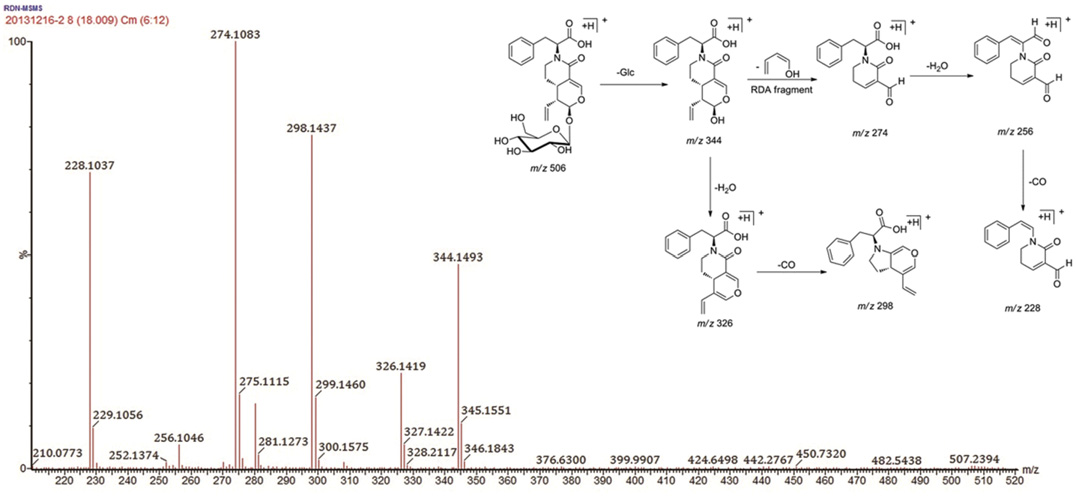
MS/MS spectra and proposed fragmentation pathways of compound 44.

Compound **44** showed a [M+H]^+^ ion at *m/z* 506.2032 with an elemental composition of C_25_H_32_NO_10_. The MS/MS spectrum of [M+H]^+^ exhibited an obvious fragment ion, [M+H-Glc]^+^, at *m/z* 344.1493 (C_19_H_22_NO_5_) from the loss of a neutral glucose residue (162 Da). The base peak at *m/z* 274.1083 (C_15_H_16_NO_4_) was formed by a retro-Diels-Alder (RDA) cleavage reaction in the aglycone moiety. This precursor ion (C_15_H_16_NO_4_) further produced two characteristic fragment ions at *m/z* 256.1046 (C_15_H_14_NO_3_) and 228.1037 (C_14_H_14_NO_2_) through the loss of one H_2_O and the further loss of one CO, respectively. Moreover, two characteristic fragment ions at *m/z* 326.1419 (C_19_H_20_NO_4_) and 298.1437 (C_18_H_20_NO_3_) were formed from [M+H-Glc]^+^ by the successive losses of H_2_O and CO. Thus, compound **44** could be tentatively identified as L-phenylalaninosecologanin B (Fig 4), which was further confirmed by comparison to a reference standard.

**Fig 5:**
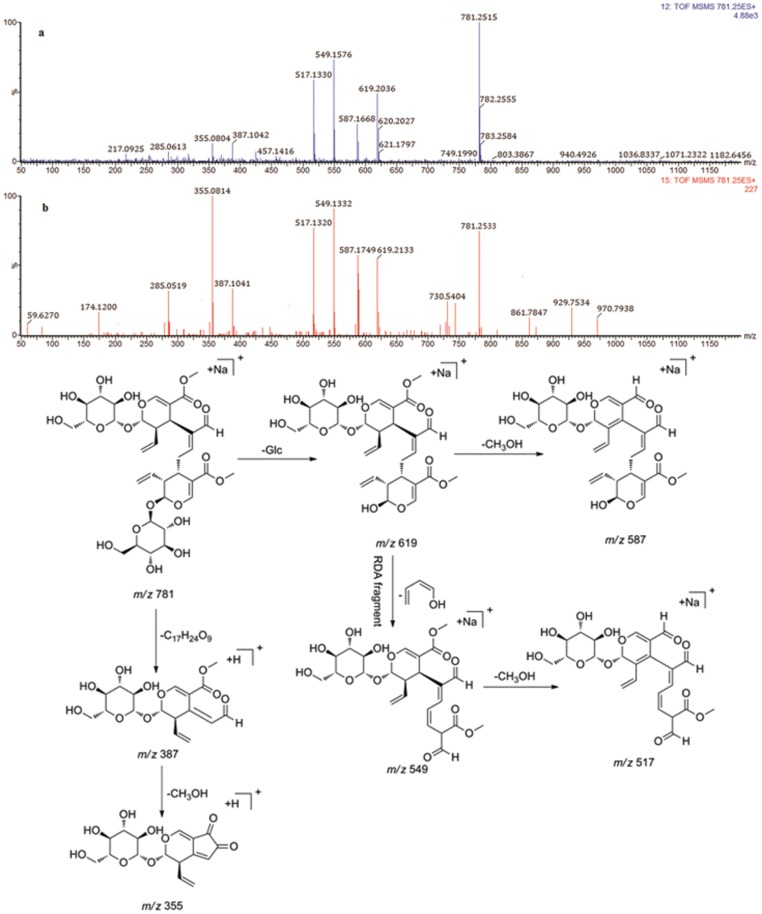
MS/MS spectra of compounds 37 (a) and 41 (b) and proposed fragmentation pathways of compound 37.

Compounds **37** and **41** gave the same molecular formula of C_34_H_46_O_19_ from their precursor [M+Na]^+^ ions at *m/z* 781.2515 and 781.2533, respectively. In (Fig 5a and 5b) illustrated the positive ion mode MS/MS spectra of compounds **37** and **41** at 35 eV and 42 eV trap collision energy, respectively. Their diagnostic fragmentation ions demonstrated minor differences with the exception for their peak intensity. For example, in compound **37**, a predominant [M+Na]^+^ ion was observed at *m/z* 781.2515 (C_34_H_46_O_19_Na, 781.1531, 2.0 ppm). An obvious fragment ion [M+Na-Glc]^+^ at *m/z* 619.2036 was observed by the neutral loss of 162 Da. The additional loss of CH_3_OH (32 Da) produced [M+Na-Glc-CH_3_OH]^+^ at *m/z* 587.1668. The second predominant peak at *m/z* 549.1576 (C_24_H_30_O_13_Na) was formed through an retro-Diels-Alder (RDA) reaction with the neutral loss of C_4_H_6_O. The fragment ion at *m/z* 517.1330 was formed by successive loss of another CH_3_OH molecule from the ion at *m/z* 549.1576. A minor peak at *m/z* 387.1042 (C_17_H_23_O_10_) was formed by the cleavage of another C_17_H_24_O_9_ fragment. Further loss of CH_3_OH (32 Da) from 387.1042 (C_17_H_23_O_10_) produced *m/z* 355.0804 (C_16_H_19_O_9_). Thus, compound **37** was tentatively identified as (*E*)-aldosecologanin. Compounds **37** and **41** were further confirmed to be (*E*)-aldosecologanin and (*Z*)-aldosecologanin, respectively, based on comparing their retention times with the isolated compounds and fragmentation pathways observed in our MS/MS experiments.

**Fig 6:**
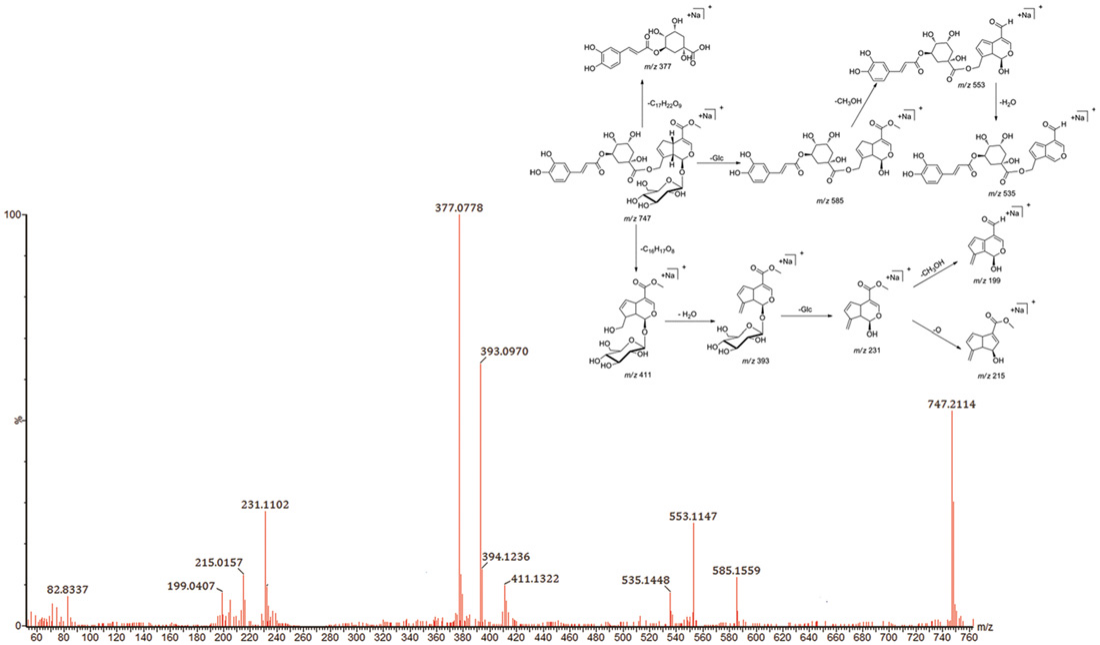
MS/MS spectra of compound 35 and proposed fragmentation pathways of compound 35.

The [M+Na]^+^ ion of compound **35** was observed at *m/z* 747.2114, indicating an elemental composition of C_33_H_40_O_18_Na (747.2115, 0.1 ppm). The MS/MS spectrum of [M+Na]^+^ showed a base peak at *m/z* 377.0778 (C_16_H_18_O_9_Na) produced by cleavage of the C_17_H_22_O_9_ fragment. In addition, neutral loss of a glucose unit (162 Da) generated the [M+Na-Glc]^+^ at *m/z* 585.1559. Successive losses of CH_3_OH and H_2_O molecules formed [M+Na-Glc-CH_3_OH]^+^ at *m/z* 553.1147 and [M+Na-Glc-CH_3_OH-H_2_O]^+^ at *m/z* 535.1448. Loss of the C_16_H_16_O_8_ fragment produced [M+Na-C_16_H_16_O_8_]^+^ at *m/z* 411.1322 (C_17_H_24_O_10_Na), and successive loss of another H_2_O molecule from 411.1322 led to the formation of an obvious ion at *m/z* 393.0970. The fragment ion at *m/z* 231.1102 (C_11_H_12_O_4_Na) was produced by neutral loss of a glucose unit (162 Da). Other characteristic fragment ions were formed, such as *m/z* 215.0157 and 199.0407, by successive or simultaneous losses of an O atom and a CH_3_OH molecule from *m/z* 231.1102 (Fig 6). Thus, compound **35** was identified as jasmigeniposide A, which was a new compound isolated from RDN. This result was further confirmed through reference standard comparison.

In addition to the above compounds, 37 iridoid glycosides (compounds **1–34**, **36**, **38** and **43**) were identified or tentatively characterized from RDN (Table 1) based on their molecular weights and the tandem fragmentation patterns.

### 3.4.2. Organic acids

According to previous research, caffeoylquinic acids as the main bioactive components in RDN were found in *L*. *japonica* Thunb., *G*. *jasminoides* Ellis and *A*. *annua* L. The structures of these typical constituents generally consist of a quinic acid moiety and mono- or dicaffeic acids that are linked to the 3-OH and/or 4-OH and/or 5-OH [22]. These compounds exhibit common proposed fragmentation pathways and diagnostic fragmentation ions, such as *m/z* 353, 191, 179, 173, 135, etc. The differences in the diagnostic fragmentation ion intensity could be used to identify their structures. As shown in (Fig 1), 15 peaks, including 14 caffeoylquinic acids and one caffeoylquinic substituted new iridoid glycoside, appeared in EIC mode and were considered as target compounds by extracting diagnostic ions 191.0556 and 179.0340 in the high-energy MS^E^ function.

**Fig 7:**
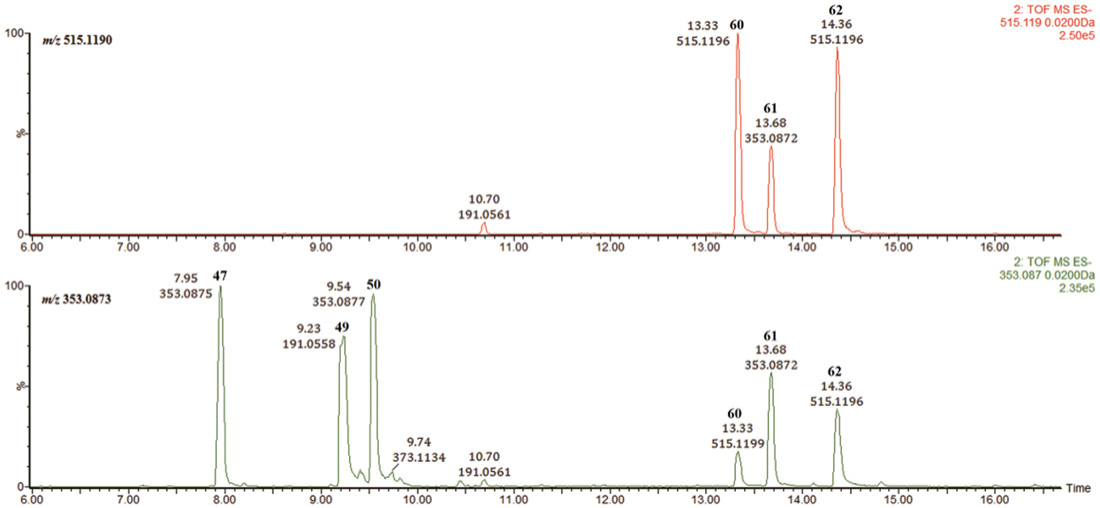
EIC-MS peaks of all possible caffeoylquinic acids in Re-Du-Ning injection.

Six peaks were easily located in the chromatogram of RDN by extracting *m/z* 353.0873. Similarly, three parent ions at *m/z* 515.1190 were located. By comparison with accurate retention times, the first three ions were assigned as monocaffeic acids, while the latter three were identified as dicaffeic acids (Fig 7). According to the literature [25–27], the linkage position of the caffeoyl groups on quinic acid could be determined according to its MS^2^ fragmentation behavior. Briefly, when the caffeoyl group was linked to 3-OH or 5-OH, the [quinic acid-H]^-^ ion at *m/z* 191 was the base peak, and the [caffeic acid-H]^-^ ion at *m/z* 179 was more significant for 3-*O*-caffeoylquinic acids. The [quinic acid-H_2_O-H]^-^ ion at *m/z* 173 was the prominent peak when the caffeoyl group was linked to 4-OH. In our experiment, this fragmentation behavior was also observed in the negative mode MS^E^ spectra. Thus, compounds **47**, **49** and **50** were identified as 5-*O*-caffeoylquinic acid, 3-*O*-caffeoylquinic acid and 4-*O*-caffeoylquinic acid, respectively. Similarly, compounds **51**, **53** and **55** were identified as 5-*O*-caffeoylquinic methyl ester, 3-*O*-caffeoylquinic methyl ester and 4-*O*-caffeoylquinic methyl ester, respectively.

Compound **61** had a base peak ion at *m/z* 191.0561 and a secondary peak at *m/z* 179.0349. As reviewed above, **61** could be identified as a 3-substituted quinic acid. Therefore, peak 18 was identified as 3,5-di-*O*-caffeoylquinic acid, which was further confirmed by comparison to a reference standard. Compounds **60** and **62** both produced a base peak at *m/z* 173, indicating that they were both 4-substituted quinic acids. According to literature [28], the retention time of 3,4-di-*O*-caffeoylquinic acid is shorter than that of 4,5-di-*O*-caffeoylquinic acid, and thus, the compounds were identified as 3,4-di-*O*-caffeoylquinic acid and 4,5-di-*O*-caffeoylquinic acid, respectively. These retention times were consistent with those of the separate compounds. In addition, compounds **63**, **64** and **66** were identified as 3,4-di-*O*-caffeoylquinic methyl ester, 3,5-di-*O*-caffeoylquinic methyl ester and 4,5-di-*O*-caffeoylquinic methyl ester, respectively.

Compound **46** was identified as quinic acid by comparison with a standard compound. Compounds **54, 57** and **59** were tentatively assigned as 3-hydroxy-4-methoxy styrene acrylic acid, 3-*O*-caffeoylshikimic acid and syringaldehyde, respectively, by matching their accurate molecular weights with those in a chemical database. These assignments were further corroborated by comparison with standard substances. Compounds **48**, **52**, **56**, **58** and **66** were identified by comparison with isolated compounds from RDN (as listed in Table 1).

### 3.4.3. Flavonoids

The MS/MS behaviors of flavonoids and their glycosides have been extensively described [22, 29–31]. Briefly, the primary MS/MS behavior of aglycones was described by the RDA fragmentation pathway. Successive loss of CO from the ketone group, C-fragmentation and loss of radicals, such as CH_3_ and CHO, have been described. For flavonoid glycosides, the glycosidic bond is easily cleaved in positive ion mode, and the neutral loss of 162 Da is the characteristic fragment ion of flavonoid *O*-glycosides. The fragment ion at [M+H-308]^+^ corresponds to the loss of a rutinose unit.

As shown in Table 1, a total of nine flavonoids were screened from RDN, four of which were unambiguously identified as rutin (**67**), hyperoside (**68**), luteolin-7-*O*-β-d-glucoside (**70**), luteolin (**73**) and quercetin (**74**) by comparison with standard constituents isolated from RDN. The other four flavonoids were tentatively identified as lonicerin (**69**), eriodictyol (**71**), artemetin (**72**) and eupatin (**75**) by matching their extract molecular weights with the chemical database and MS/MS fragmentation behavior.

### 3.4.4. Identification of other compounds

Another 15 obvious peaks in the extracted ion chromatogram of RDN were identified (Table 1). Three coumarins, compounds **87**, **88** and **89**, were unambiguously identified as scopoletin, 7-hydroxy-6,8-dimethoxyphenyl coumarin and coumarin, respectively, by comparison with the isolated reference standards. Among the four sesquiterpenes, compound **83** was tentatively assigned as 7,8,11-trihydroxyguai-4-en-3-one 8-*O*-β-d-glucopyranoside by matching its mass with the chemical database within 5 ppm. Compounds **84**, **85** and **86** were confirmed by matching to the retention times of the isolated reference standards. Similarly, seven lignans (compounds **76**, **77**, **78**, **79**, **80**, **81** and **82**) and one monoterpene (compound **90**) were also identified.

### Conclusion

In this work, an approach involving UPLC-ESI-Q/TOF-MS coupled with MS^E^ data acquisition was developed to profile multiple chemical constituents in RDN. Diagnostic ions were used as invaluable markers for the screening of target compounds. A total of 53 compounds, including two new iridoids, were identified or tentatively characterized using this method. Due to the structural complexity of the chemical constituent types in TCMPs, the present analytical approach still has a limitation in the detection of low-abundance components. The remaining 37 compounds were identified according to their accurate mass measurements within 5 ppm error, tandem MS behaviors, database-matching and reference standards. The RDN herbal sources were unambiguously confirmed by comparing the extracted ion chromatograms (EICs) of RDN and ingredient herbal extracts. The results of our study not only provide a certain foundation for further studies of RDN but also demonstrate chemical profile analyses of TCMPs via UPLC-ESI-Q/TOF-MS and diagnostic ion screening using MS^E^.

## Supporting Information

S1 Fig Total ion chromatograms (TIC) of three different types of 50 mm columns. (DOCX)

S2 Fig MS chromatograms of diagnostic ions: (A) EICs of diagnostic ions 209.0814, 251.0532, 235.0582 and 215.0320 in the high-energy function of MS^E^; (B) TIC of RDN in the high-energy function of MS^E^; (C) EICs of diagnostic ions 209.0814, 251.0532, 235.0582 and 215.0320 in the low-energy function of MS^E^; (D) TIC of RDN in the MS^E^ low-energy function.
(DOCX)

S3 Fig MS chromatograms of diagnostic ions: (A) EICs of diagnostic ions 285.0399 and 301.0348 in the high-energy function of MS^E^; (B) TIC of RDN in the high-energy function of MS^E^; (C) EICs of diagnostic ions 285.0399 and 301.0348 in the low-energy function of MS^E^; (D) TIC of RDN in the MS^E^ low-energy function.
(DOCX)

S4 Fig Structures of identified components in RDN (Red: new compounds) (DOCX)

S5 Fig Basic peak intensity (BPI) profiles of three individual herbs in positive ion mode. Gj = *Gardenia jasminoides* Ellis, Lj = *Lonicera japonica* Thunb. and Aa = *Artemisia annua* L.
(DOCX)

S6 Fig Basic peak intensity (BPI) profiles of three individual herbs in negative ion mode. Gj = *Gardenia jasminoides* Ellis, Lj = *Lonicera japonica* Thunb. and Aa = *Artemisia annua* L.
(DOCX)

S7 Fig Graphical abstract of our research. (DOCX)
