# Supplementary material for: Chemical Profiling of Re-Du-Ning Injection by Ultra-Performance Liquid Chromatography Coupled with Electrospray Ionization Tandem Quadrupole Time-of-Flight Mass Spectrometry through the Screening of Diagnostic Ions in MSE Mode
Source: PLoS One. 2015 Apr 13;10(4):e0121031. doi: 10.1371/journal.pone.0121031 (PMC4395252; doi:10.1371/journal.pone.0121031)
Supplement: S3 Fig — (A) EICs of diagnostic ions 285.0399 and 301.0348 in the high-energy function of MSE; (B) TIC of RDN in the high-energy function of MSE; (C) EICs of diagnostic ions 285.0399 and 301.0348 in the low-energy function of MSE; (D) TIC of RDN in the MSE low-energy function. (DOCX) [file pone.0121031.s003.docx]

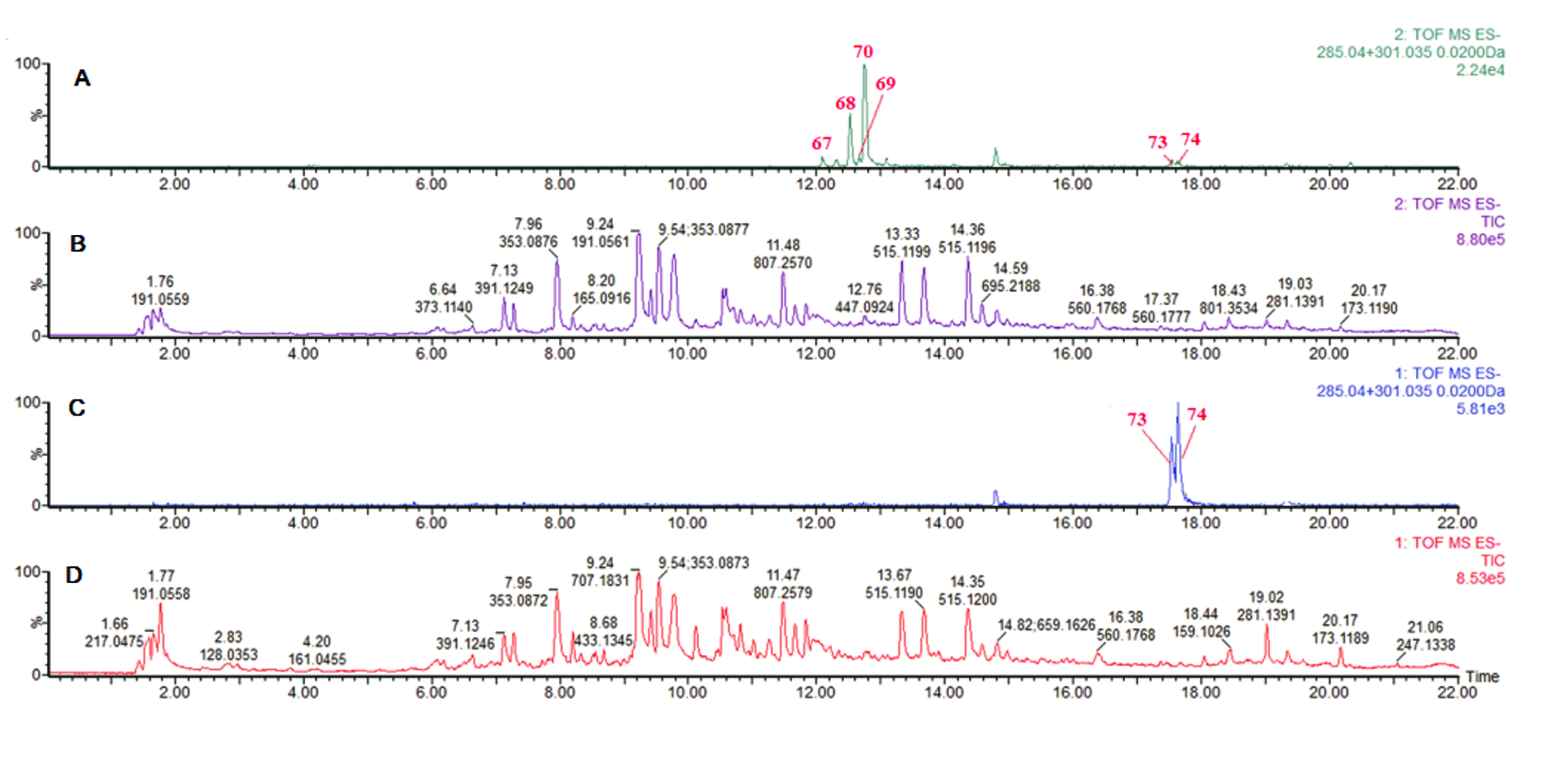


**S3 Fig. MS chromatograms of diagnostic ions**: (A) EICs of diagnostic ions 285.0399 and 301.0348 in the high-energy function of MS^E^; (B) TIC of RDN in the high-energy function of MS^E^; (C) EICs of diagnostic ions 285.0399 and 301.0348 in the low-energy function of MS^E^; (D) TIC of RDN in the MS^E^ low-energy function.
