# Supplementary material for: Chemical Profiling of Re-Du-Ning Injection by Ultra-Performance Liquid Chromatography Coupled with Electrospray Ionization Tandem Quadrupole Time-of-Flight Mass Spectrometry through the Screening of Diagnostic Ions in MSE Mode
Source: PLoS One. 2015 Apr 13;10(4):e0121031. doi: 10.1371/journal.pone.0121031 (PMC4395252; doi:10.1371/journal.pone.0121031)
Supplement: S6 Fig — Gj = Gardenia jasminoides Ellis, Lj = Lonicera japonica Thunb. and Aa = Artemisia annua L. (DOCX) [file pone.0121031.s006.docx]

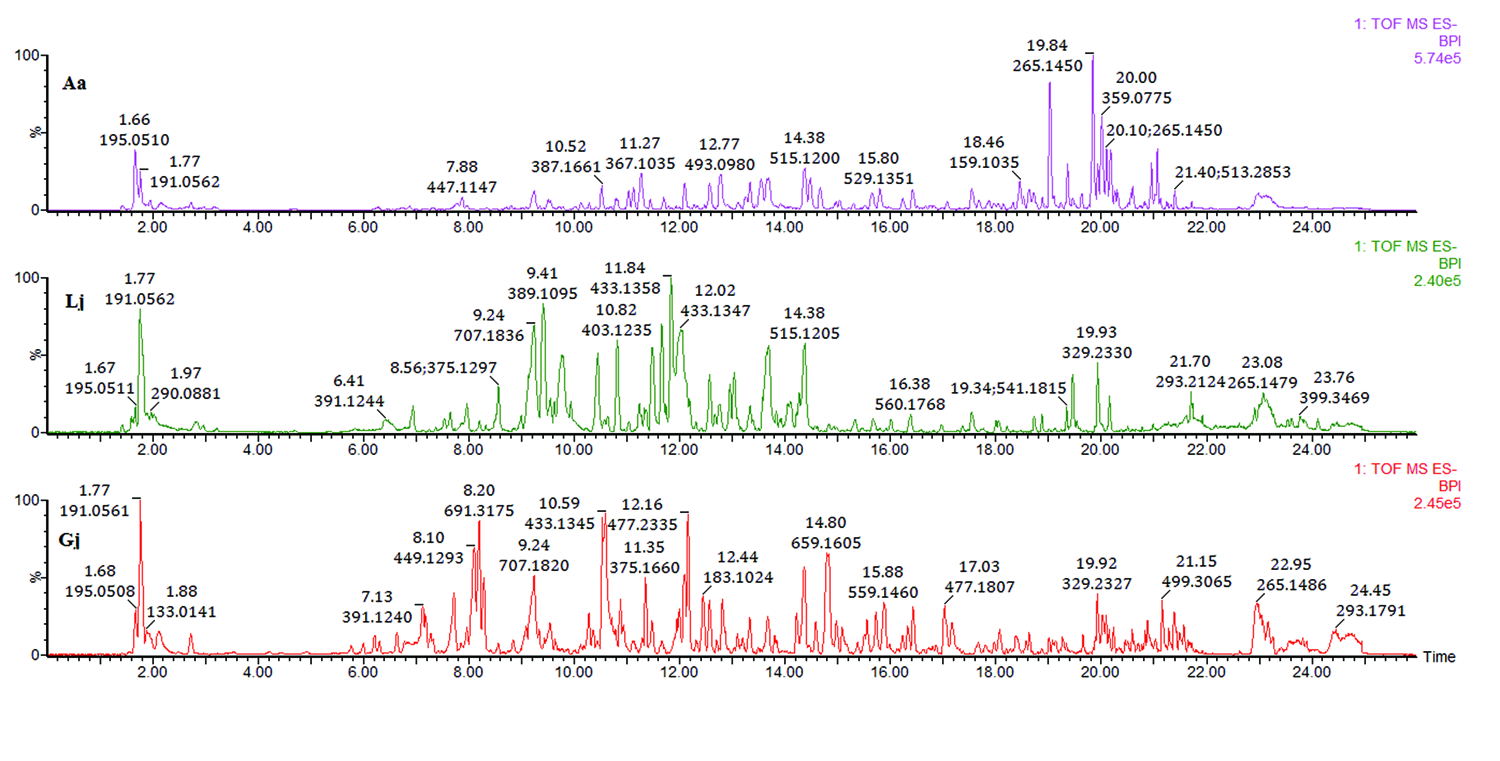


**S6 Fig. Basic peak intensity (BPI) profiles of three individual herbs** in negative ion mode. Gj = *Gardeniajasminoides* Ellis, Lj = *Lonicera japonica* Thunb. and Aa = *Artemisia annua* L.
