# Supplementary figures and images for: Chemical Profiling of Re-Du-Ning Injection by Ultra-Performance Liquid Chromatography Coupled with Electrospray Ionization Tandem Quadrupole Time-of-Flight Mass Spectrometry through the Screening of Diagnostic Ions in MSE Mode
Source: PLoS One. 2015 Apr 13;10(4):e0121031. doi: 10.1371/journal.pone.0121031 (PMC4395252; doi:10.1371/journal.pone.0121031)

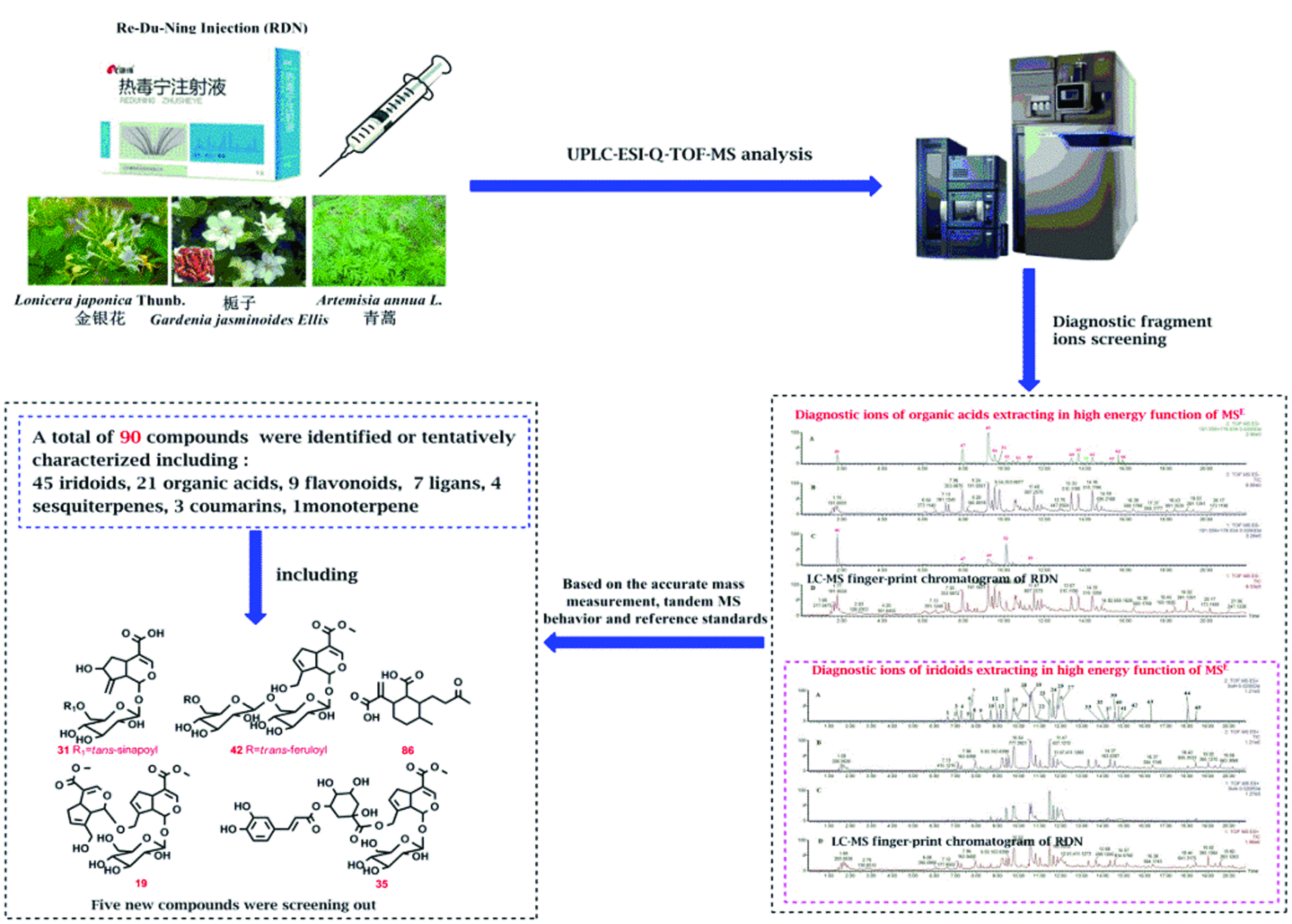


**S7 Fig. Graphical abstract** of our research.

Supplement: S7 Fig — (DOCX) [file pone.0121031.s007.docx]
